# Identification of Energy Metabolism Genes for the Prediction of Survival in Hepatocellular Carcinoma

**DOI:** 10.3389/fonc.2020.01210

**Published:** 2020-08-13

**Authors:** Qinjunjie Chen, Fengwei Li, Yuzhen Gao, Gaoran Xu, Leilei Liang, Jingchao Xu

**Affiliations:** ^1^Department of General Surgery, The Second Affiliated Hospital of Dalian Medical University, Dalian, China; ^2^Department of Hepatic Surgery, The Eastern Hepatobiliary Surgery Hospital, Second Military Medical University, Shanghai, China; ^3^Department of Molecular Diagnosis, Clinical Medical College, Yangzhou University, Jiangsu, China; ^4^Department of Surgery, Tongren Hospital, Shanghai Jiao Tong University School of Medicine, Shanghai, China

**Keywords:** hepatocellular carcinoma, energy metabolism, risk model, prognosis, bioinformatics

## Abstract

Hepatocellular carcinoma (HCC) samples were clustered into three energy metabolism-related molecular subtypes (C1, C2, and C3) with different prognosis using the gene expression data from The Cancer Genome Atlas (TCGA) and Gene Expression Omnibus (GEO). HCC energy metabolism-related molecular subtype analysis was conducted based on the 594 energy metabolism genes. Differential expression analysis yielded 576 differentially expressed genes (DEGs) among the three subtypes, which were closely related to HCC progression. Six genes were finally selected from the 576 DEGs through LASSO-Cox regression and used in constructing a six-gene signature-associated prognostic risk model, which was validated using the TCGA internal and three GEO external validation cohorts. The risk model showed that high *ANLN, ENTPD2, TRIP13, PLAC8*, and *G6PD* expression levels were associated with bad prognosis, and high expression of *ADH1C* was associated with a good prognosis. The validation results showed that our risk model had a high distinguishing ability of prognosis in HCC patients. The four enriched pathways of the risk model were obtained by gene set enrichment analysis (GSEA) and found to be associated with the tumorigenesis and development of HCC, including the cell cycle, Wnt signaling pathway, drug metabolism cytochrome P450, and primary bile acid biosynthesis. The risk score calculated from the established risk model in 204 samples and other clinical characteristics were used in building a nomogram with a good prognostic prediction ability (C-index = 0.746, 95% CI = 0.714–0.777). The area under the curves (AUCs) of the nomogram model in 1-, 2-, and 3-years were 0.82, 0.77, and 0.79, respectively. Then, qRT-PCR and immunohistochemistry were used to validate the mRNA expression levels of the six genes, and significant differences in mRNA and gene expression were observed among the tumor and adjacent tissues. Overall, our study divided HCC patients into three energy metabolism-related molecular subtypes with different prognosis. Then, a risk model with a good performance in prognostic prediction was built using the TCGA dataset. This model can be used as an independent prognostic evaluation index for HCC patients.

## Introduction

Hepatocellular carcinoma (HCC) is the most common primary liver cancer and has been an important medical problem worldwide. HCC is the sixth most common malignancy in the world and the fourth leading cause of cancer-related death. HCC has been identified as the leading cause of death in patients with cirrhosis, and its incidence is expected to increase in the future (1).

Although many studies have explored the molecular, cellular, and environmental mechanisms that contribute to the occurrence of HCC, limited options are presently available for clinicians in delaying tumor progression and prolonging patients' life expectancy. Besides, the high recurrence rate and invasiveness of HCC severely limit the improvement of the curative effect of HCC, and the main mechanism of this feature is not fully understood (2). Therefore, an effective prediction model is needed for the accurate assessment of the patient's prognosis.

Energy metabolism has long been a hallmark of cancer cells, enabling tumor cells to produce adenosine triphosphate (ATP) to maintain the reduction-oxidation balance and macromolecular biosynthesis processes required for cell growth, proliferation, and migration (3, 4). Many cancers have been known to limit their energy metabolism mainly to glycolysis, producing large amounts of lactic acid even in the presence of oxygen through the Warburg effect (5). Glucose is the main source of energy for cancer cells. Compared with normal cells, tumor cells prefer the incomplete non-oxidative metabolism of glucose (6). These metabolic features promote the survival, proliferation, and metastasis of cancer cells (7). Therefore, a deeper understanding of energy metabolism in HCC can provide an important solution for the development of novel therapies. In recent years, gene chips and high-throughput sequencing have made great progress and development, which means that energy metabolism gene signatures at the mRNA level are available to predict overall survival (OS) in HCC.

In the present study, genes related to energy metabolism were collected. Gene expression data from The Cancer Genome Atlas (TCGA) and Gene Expression Omnibus (GEO) were used in constructing HCC molecular subtypes based on genes related to energy metabolism. The relationship between molecular subtypes and prognosis was further evaluated. Six genes were finally selected from the 576 differentially expressed genes (DEGs) through differential expression analysis and LASSO-Cox regression for the construction of a prognostic risk model. The risk model can evaluate the prognosis of HCC patients and be validated by the TCGA internal and GEO external validation cohorts. This model can be used as an independent prognostic evaluation index for HCC patients.

## Materials and Methods

### Data Download and Preprocessing

The **TCGA-LIHC** dataset consisted of the RNA-seq data of 371 HCC and 53 adjacent normal samples and related clinical characteristics and was downloaded from the TCGA database (https://portal.gdc.cancer.gov/). **GSE76427**, **GSE15654**, and **GSE14520** contained the gene expression data of 115, 65, and 221 HCC biopsy specimens with clinical characteristics and were downloaded from the GEO database (http://ncbi.nlm.nih.gov/geo/), respectively. The raw data were preprocessed with the following criteria: (1) the genes were excluded if the FPKM value (Fragments per Kilobase Million) was zero in more than half of the samples; (2) genes with missing expression values in more than 30% of samples were removed; (3) the invariant genes (i.e., same expression value across all samples) and low-variation genes were filtered; (4) samples without related clinical data or OS <30 days were removed; (5) normal tissue samples were removed. The **TCGA-LIHC** dataset was randomly divided into two cohorts: training cohort (*n* = 204) and internal validation dataset (*n* = 138), and the three GEO datasets were regarded as the external validation cohorts. As the data were open-access, therefore, the ethical approval by an ethics committee was not required. Human metabolic-related pathways were downloaded from the Reactome database (https://reactome.org/). A total of 594 genes related to energy metabolism were sorted out from 11 metabolic pathways (Tables S1, S2).

### Energy Metabolic Molecular Subtypes

HCC energy metabolism-related molecular subtype analysis was conducted based on the 594 genes that were sorted out above. The non-negative matrix factorization (NMF) consensus cluster in the R package “NMF” was used to cluster all the HCC samples in the TCGA-LIHC dataset. The clinicopathological characteristics of different groups were compared, and the immune scores of the subtypes were obtained by TIMER (tumor immune estimation resource) tool.

### DEG Identification and Bioinformatics Analysis

The R package of “DESeq2” was used to calculate the DEGs of the subtypes (FDR < 0.05 and |log2FC| > 1). GO and KEGG functional enrichment analyses were conducted based on the DEGs. Protein-protein interaction (PPI) analysis was conducted based on the STRING database (https://string-db.org/). The fast greedy clustering algorithm of the STRINGdb tool was used to cluster the interaction network.

### Risk Model Construction and Validation

The expression data of the DEGs in the training cohort were used in constructing a risk score model. The impact of each DEG on the OS of HCC patients was estimated by the univariate Cox proportional risk regression model. Log-rank *P* < 0.01 was considered statistically significant. Then, a LASSO-Cox regression was employed to narrow the number of genes in our model. A risk score model was established by including individual normalized gene expression values weighted by their LASSO-Cox coefficients. Internal and external validation cohorts were then used to verify the robustness of the risk model. The risk score of each sample was calculated based on our formula, and the risk score distribution was plotted by the R package of “timeROC.” Then, the samples were divided into high- and low-risk groups by a cutoff value that was calculated using the Gordon index. A log-rank test was used in comparing the survival difference between the two groups. The OS of each group was performed using the Kaplan–Meier (KM) survival curve.

### Gene Set Enrichment Analysis (GSEA)

GSEA enrichment in the TCGA-LIHC dataset was conducted for the analysis of the significantly enriched pathways in the high- and low-risk groups. c2.cp.kegg.v6.0 symbols were selected for our analysis, which included the KEGG pathways database.

### Sample Collection

HCC and adjacent tissues were collected from 44 patients (all participants were older than 16 years). None of the hepatocellular cancer patients received preoperative anti-tumor therapies. Patients and their families in this study have been fully informed and informed consent was obtained from the participants. This study was approved by the Ethics Committee of The Second Affiliated Hospital of Dalian Medical University.

### RNA Isolation and qRT-PCR Analysis

Forty-four pairs of HCC and adjacent nontumor tissues were obtained from The Second Affiliated Hospital of Dalian Medical University, Dalian, China. qRT-PCR experiments were performed to validate the mRNA expression levels of the six genes screened above. Total RNA from the HCC and adjacent normal liver tissue specimens was extracted using TRIzol reagent (Invitrogen, Thermo Scientific, Shanghai, China). Then, RNA was reverse-transcribed into cDNA with the QuantiTect reverse transcription kit (QIAGEN, Valencia, CA, USA). Real-time PCR analyses were quantified with SYBR-Green (Takara, Otsu, Shiga, Japan), The amplification conditions were as follows: 50°C for 90 s, 95°C for 10 min, 40 cycles at 95°C for 15 s, and 60°C for 1 min. The final products of PCR were analyzed based on melting curves. Each RNA sampling was performed in triplicate, and the RNA expression levels of the genes were calculated relative to that of GAPDH.

### Immunohistochemistry

Each group of HCC samples was fixed in 10% formalin, embedded in paraffin, and processed as 5 μm continuous sections. Samples were dewaxed with discontinuous concentrations of ethanol and blocked to inhibit endogenous peroxidase. They were then heated in a microwave to retrieve antigens, cooled to room temperature, and then blocked by incubation in goat serum for 30 min at 37°C. Samples were incubated in rabbit anti-ANLN, anti-ENTPD2, anti-TRIP13, anti-PLAC8, anti-G6PD and anti-ADH1C (Abcam, Cambridge, UK; 1:1,200) overnight at 4°C, followed by incubation with horseradish peroxidase-coupled goat anti-rabbit secondary antibody at 37°C for 30 min and stained using 3,3′-diaminobenzidine. The cell nucleus was stained blue by hematoxylin. Sections were then dehydrated, cleared by xylene, and mounted. *ANLN, ENTPD2, TRIP13, PLAC8, G6PD*, and *ADH1C* expression were detected by IHC using a streptavidin peroxidase method. *ANLN, ENTPD2, TRIP13, PLAC8, G6PD*, and *ADH1C* expression in the liver were used as a positive control. Samples incubated with PBS instead of the *ANLN, ENTPD2, TRIP13, PLAC8, G6PD*, and *ADH1C* primary antibody were used as a negative control. Positive and negative controls were included for each batch of immunohistochemically stained sections. The experimental procedure was performed according to strict adherence to the manufacturers' instructions.

### Statistical Analysis

R software (version 3.6.1) and SPSS software (version 23.0) were used to complete all the statistic work. Multiple imputation analyses were used for missing values (8). Bootstrap sampling was assigned 100-fold so that random assignment bias that can affect the stability of the subsequently built risk model was prevented and the distribution of covariates, such as age and tumor Gleason grade, was ensured. TNM staging was the same between the randomly selected samples and all the samples. The differences between categorical variables were compared by using the Chi-square test. Student's *t*-test was used for comparison of quantitative values according to normal distribution. The Mann–Whitney *U*-test was utilized for the comparison of non-normal distributed quantitative data. OS was calculated by the KM method, and the differences between the groups were compared by using the log-rank test. Cox proportional hazard model was used to analyze the significant factors affecting OS. *P* < 0.05 was considered statistically significant.

## Results

### Patients' Characteristics

**Figure 1:**
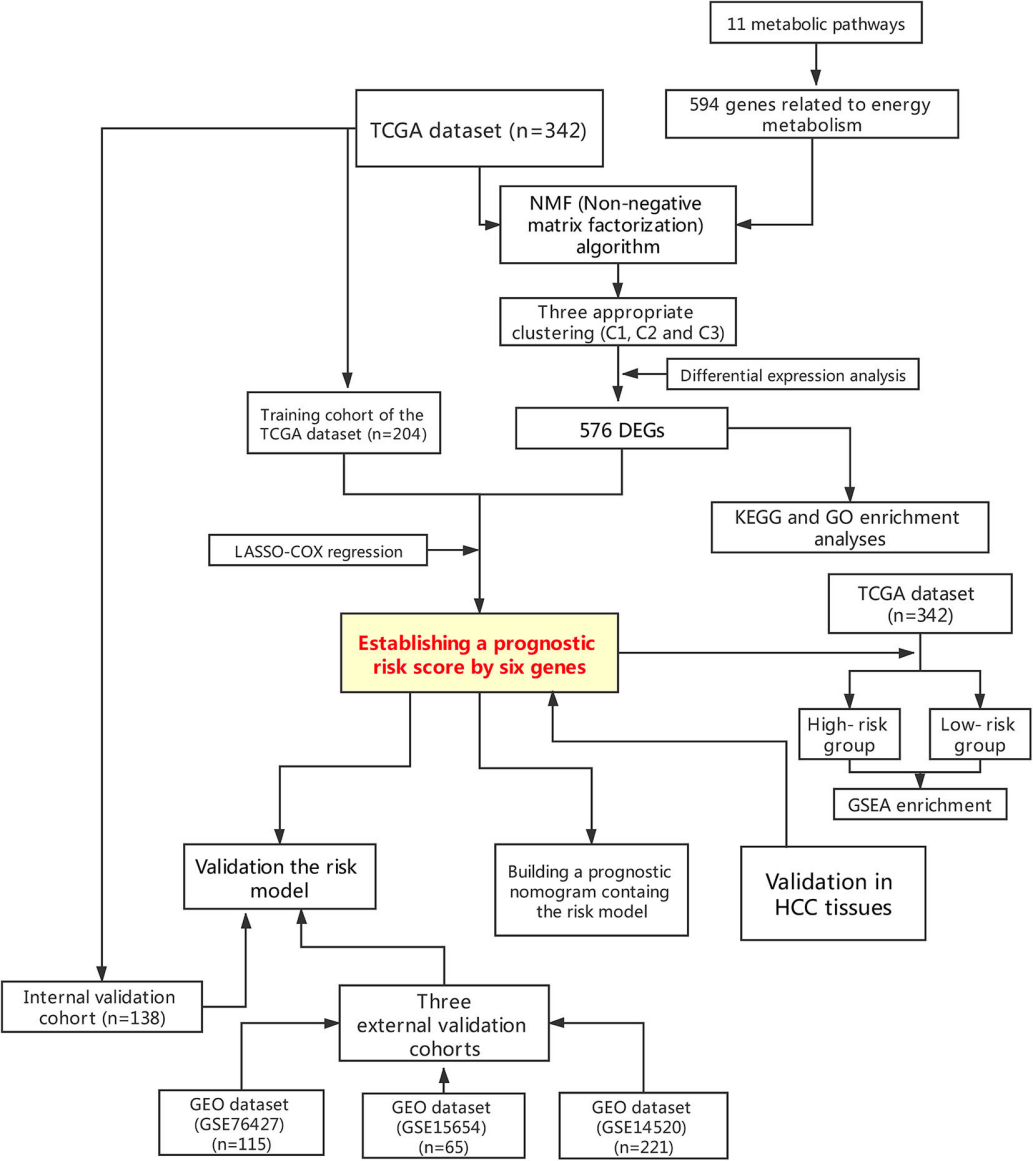
Flowchart presenting the process of establishing the gene signature and prognostic nomogram of pancreatic cancer in this study.

This study was conducted according to the flow chart shown in Figure 1. A total of 342 HCC samples from the **TCGA-LIHC** dataset and a total of 401 samples from the **GSE76427**, **GSE15654**, and **GSE14520** datasets, respectively, were screened for further analyses. The clinical characteristics of the TCGA training cohort (*n* = 204) and the internal validation cohort (*n* = 138) are shown in Table 1. The characteristics included age, gender, existing the inducement to HCC, hepatitis virus infection, Eastern Cooperative Oncology Group (ECOG) score, T stage, N stage, M stage, tumor Gleason grade, and survival status. No statistically significant differences (*P* > 0.05) were observed between the training cohort and the internal validation cohort, indicating that they were comparable.

**Table 1:** Clinicopathological characteristics between training and internal validation cohorts.

**Characteristics**		**TCGA training cohort (*n* = 204)**	**TCGA internal validation cohort (*n* = 138)**	***P*-value**
Age (years)	≤50	43	30	0.894
	>50	161	108	
Gender	Female	62	47	0.475
	Male	142	91	
Inducement	Yes	143	98	0.855
	No	61	40	
Hepatitis virus infection	Yes	89	52	0.273
	No	115	86	
ECOG score	<2	192	127	0.452
	≥2	12	11	
Pathologic T	T 1	95	73	0.168
	T 2	48	36	
	T 3	48	26	
	T 4	11	2	
Pathologic N	N 0	148	91	0.250
	N 1	1	2	
	N X	54	45	
Pathologic M	M 0	148	96	0.550
	M 1/ M X	56	42	
Tumor TNM stage	Stage I	93	68	0.717
	Stage II	44	33	
	Stage III	52	28	
	Stage IV	2	1	
Gleason grade	G1	31	22	0.979
	G2	95	66	
	G3	66	45	
	G4	8	4	
Survival status	Alive	128	91	0.546
	Dead	76	47	

*TNM stage, ACJJ 6^TH^ or 7^TH^ TNM stage; Inducement, factors associated with the occurrence of HCC; Hepatitis virus, Hepatitis B and/or hepatitis C viruses; ECOG, Eastern Cooperative Oncology Group*.

### Energy Metabolic Molecular Subtypes

**Figure 2:**
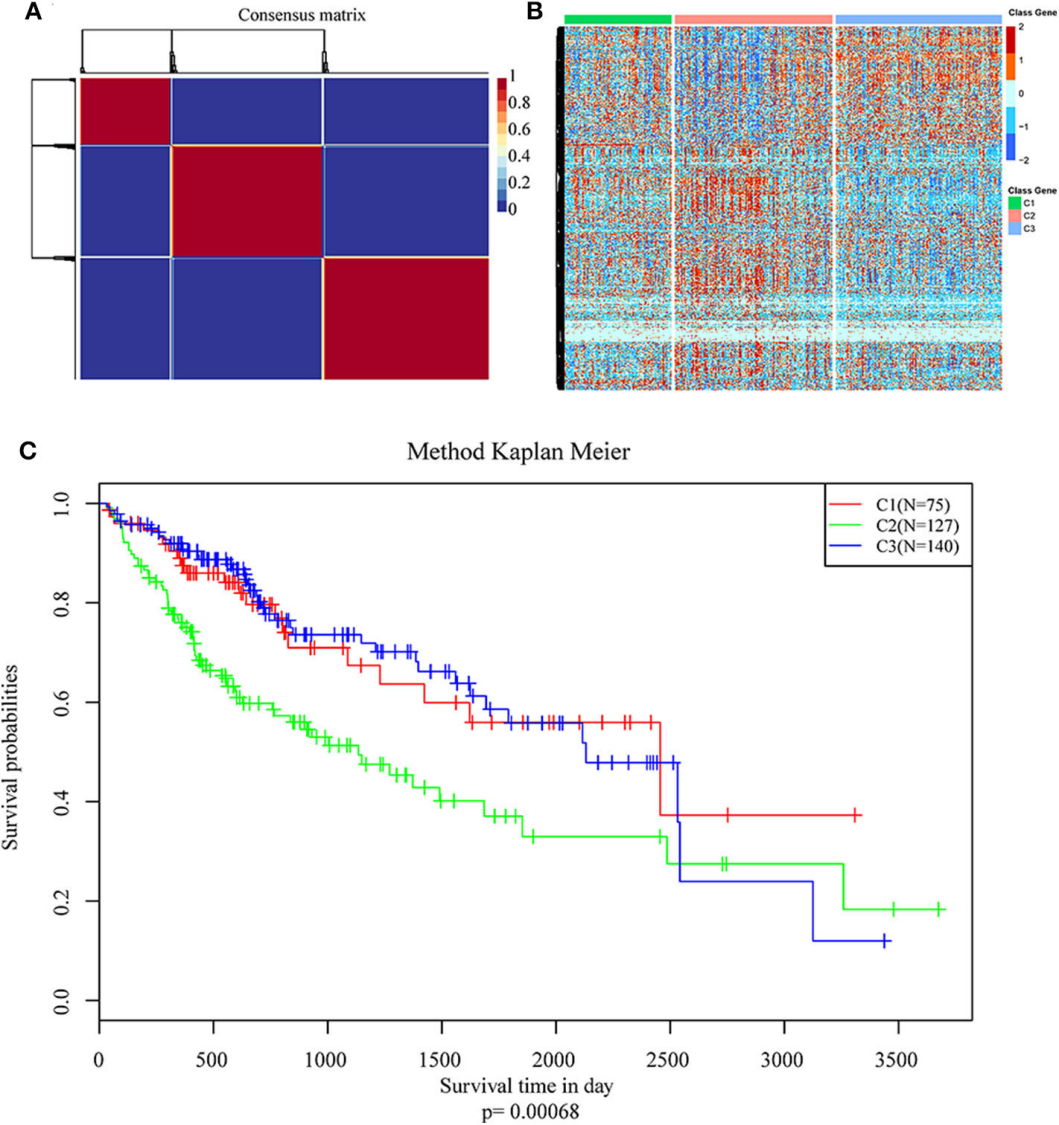
Identification of molecular subtypes of hepatocellular carcinoma (HCC) using TCGA data associated with prognosis. **(A)** The non-negative matrix factorization (NMF) consensus matrix plot for 594 metabolism-related genes identified three distinct HCC subtypes: C1, C2, and C3. **(B)** Heatmap of the expression of genes related to energy metabolism in three molecular subtypes; **(C)** Kaplan–Meier (KM) curves of overall survival (OS) in the three molecular subtypes.

NMF algorithm was used to cluster HCC samples in the **TCGA-LIHC** dataset. The optimal clustering number was selected by cophenetic, dispersion, and by using silhouette indicators. Overall, the most appropriate number of clustering was three (Figure 2A, Figure S1). The expression levels of energy metabolism-related genes seem to differ among the three subtypes (Figure 2B). The prognosis signature among them was further analyzed. The C2 subtype had the worst prognosis (Figure 2C, ***P* < 0.001**). Although C1 and C3 belonged to two different energy metabolic molecular subtypes, they had nearly the same prognosis. The clinicopathological characteristics of the three molecular subtypes were then compared. Age, gender, existing the inducement to HCC, hepatitis virus infection, ECOG score, T stage, tumor TNM stage, Gleason grade, and survival status among subtypes reached statistical significance (Table 2, ***P* < 0.05**). Furthermore, the immune scores among the subtypes were evaluated. As shown in Figure S2, all the six immune cell scores of the C2 subtype were significantly higher than those of the C1 and C3 subtypes. The C1 subtype had significantly lower CD8^+^ T cell, neutrophil, and dendritic than those of C3. However, marginal significance was shown for the macrophage cell between the C1 and C3. The results above might indicate that a complex relationship exists between the immune invasion and prognosis in HCC patients.

**Table 2:** Clinicopathological characteristics among three molecular subtypes.

**Characteristics**		**C1**	**C2**	**C3**	***P*-value**
Age (years)	≤50	12	37	24	0.031
	>50	63	90	116	
Gender	Female	10	50	49	<0.001
	Male	65	77	91	
Inducement	Yes	45	107	89	<0.001
	No	30	20	51	
Hepatitis virus infection	Yes	35	41	65	0.027
	No	35	86	80	
ECOG score	<2	65	95	120	0.033
	≥2	10	32	20	
Pathologic T	T1	32	51	85	0.005
	T2	20	35	29	
	T3	21	36	17	
	T4	1	5	7	
Pathologic N	N0	53	97	89	0.240
	N1	0	3	0	
Pathologic M	M0	55	97	92	0.616
	M1/MX	0	1	2	
Tumor TNM stage	I	30	50	81	0.003
	II	19	31	27	
	III	21	41	18	
	IV	0	1	2	
Gleason grade	G1	17	8	28	<0.001
	G2	33	55	73	
	G3	21	54	36	
	G4	2	8	2	
Survival status	Alive	54	66	99	0.002
	Dead	21	61	41	

*TNM stage, ACJJ 6^TH^ or 7^TH^ TNM stage; Inducement, factors associated with the occurrence of HCC; Hepatitis virus, Hepatitis B and/or hepatitis C viruses; ECOG, Eastern Cooperative Oncology Group*.

### DEG Identification and Bioinformatics Analysis

**Figure 3:**
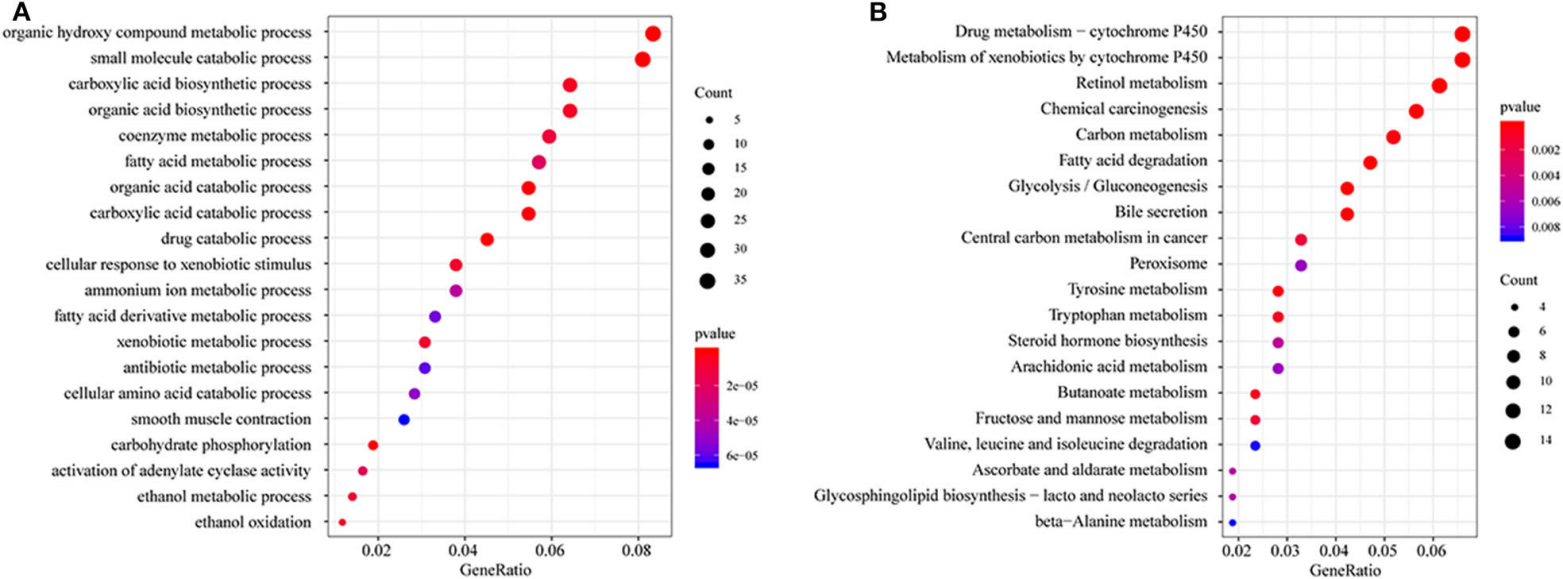
Functional enrichment analysis of differentially expressed genes (DEGs). **(A)** GO enrichment analysis results, showing only the first 20 terms; **(B)** KEGG pathway enrichment analysis results, showing only the first 20 pathways. GeneRatio: refers to the ratio of the number of genes enriched in the term/pathway to the total number of genes in the term/pathway.

All DEGs between C1/C3 and C2 were identified with the above criteria (Figure S3, Tables S3, S4). A total of 576 DEGs were identified (Table S5). Homogeneity tests between subtypes were performed. The results are shown in Figures S4, S5. Then, GO and KEGG enrichment analyses were performed. The DEGs were mainly enriched in organic hydroxy compound metabolic, small molecule catabolic, carboxylic acid biosynthetic, and organic acid biosynthetic processes (Figure 3A) and pathways related to the metabolism of xenobiotics by cytochrome P450, retinol metabolism, chemical carcinogenesis carbon metabolism, and fatty acid degradation (Figure 3B). The enrichment results are listed in Tables S6, S7. Furthermore, a PPI network was built based on the 576 DEGs. The genes were significantly clustered into eight networks (Figure S6, Table S8).

### Establishing a Prognostic Risk Model and the HCC Tissue Validation

**Figure 4:**
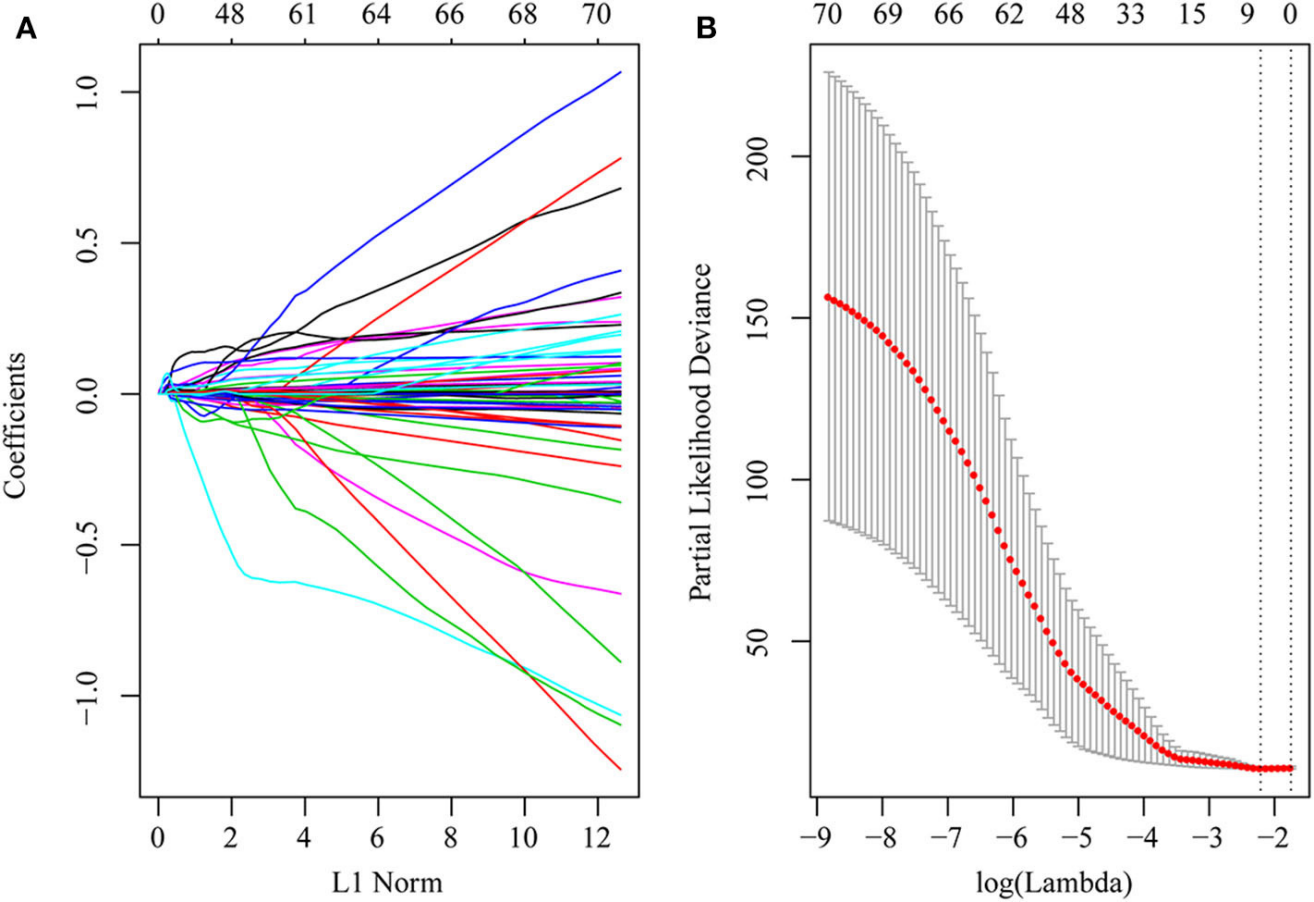
DEGs in univariate Cox regression with *P*-value of <0.01 were included in the LASSO-Cox regression model for variable compression to establish an optimal prognostic model. The model with lambda = 0.1093823 was selected as the final model. **(A)** LASSO-Cox regression coefficient selection and variable screening. The lower horizontal axis represents lambda value, and the upper horizontal axis scale represents the number of variables in the lasso-cox regression model, the regression coefficient (x) of which is not 0. The left vertical axis represents the value of the regression coefficient (x). **(B)** Cross-validation in the LASSO-Cox regression model to select the tuning parameter. The horizontal axis represents the log (lambda) value, and the vertical axis represents partial likelihood deviance. The red dots in the figure represent partial likelihood deviations ± standard error for different tuning parameters.

First, the risk score model was constructed based on the training cohort. Univariate Cox regression was performed on each DEG. A total of 71 genes were identified as risk factors for the OS of HCC patients (Table S9). Then, LASSO-Cox regression analysis was used to narrow the number of genes for the establishment of our risk score model. The model with a lambda of 0.1093823 was selected as the final model, which contained six genes (Figure 4). The model formula was as follows:

$$\begin{array}{l} \text{Risk score}=\text{0.011}*\text{ANLN}+\text{0.014}*\text{ENTPD2}+\text{0.001}*\text{TRIP13}\\ \;\text{+0.006}*\text{PLAC8}+\text{0.001}*\text{G6PD}-\text{2.037881e}\\ \;-\text{06}*\text{ADH1C} \end{array}$$

As shown in the above formula, the high expression levels of *ANLN, ENTPD2, TRIP13, PLAC8*, and *G6PD* as risk factors of prognosis were associated with high risk. However, the high expression of *ADH1C* as a protective factor of prognosis was associated with low risk.

**Figure 5:**
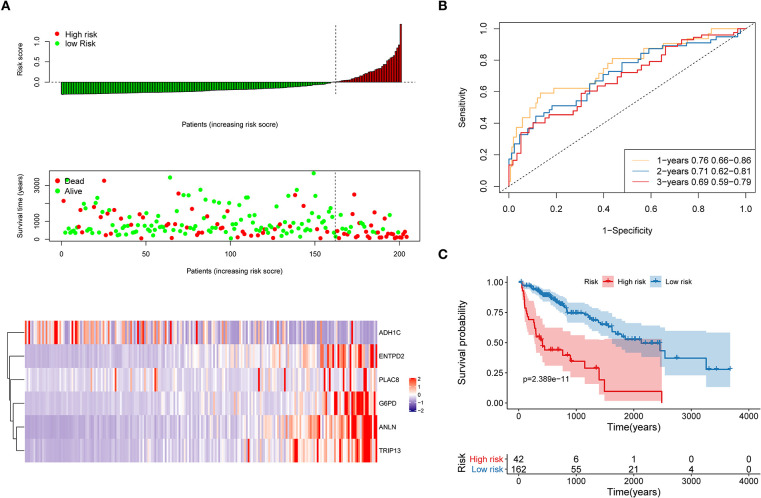
Evaluation of the performance of the risk model in the training cohort. **(A)** Distribution of risk score, OS, survival status (red dots indicate death, blue dots indicate alive) and the six genes expression heat map in the training cohort; **(B)** ROC curves and area under the curve (AUC) for 1-, 2-, and 3-year survival in the training cohort of the risk model; **(C)** Kaplan–Meier (KM) curves of the OS in the training cohort.

The distribution of each patient's risk score was obtained after the calculation of the risk score of each patient in the training cohort, as shown in Figure 5. A positive correlation between risk score and the event of death was likely present (Figure 5A). The formula's prognosis prediction efficiency was analyzed for 1, 2, and 3 years, as shown in Figure 5B. The model had a relatively high value of area under the curve (AUC), which was above 0.69. Furthermore, patients in the training cohort were divided into high- and low-risk groups based on their risk scores. The optimal cutoff value was selected by using the Gordon index (cutoff = 0.334235), and KM curves of survival were also performed in Figure 5C. A significant difference in survival probability between the two groups (*P* < 0.001) was observed, as shown in Figure 5C. Patients with high-risk scores were associated with significantly worse OS, thereby suggesting that the high-risk score was an adverse prognostic factor.

**Figure 6:**
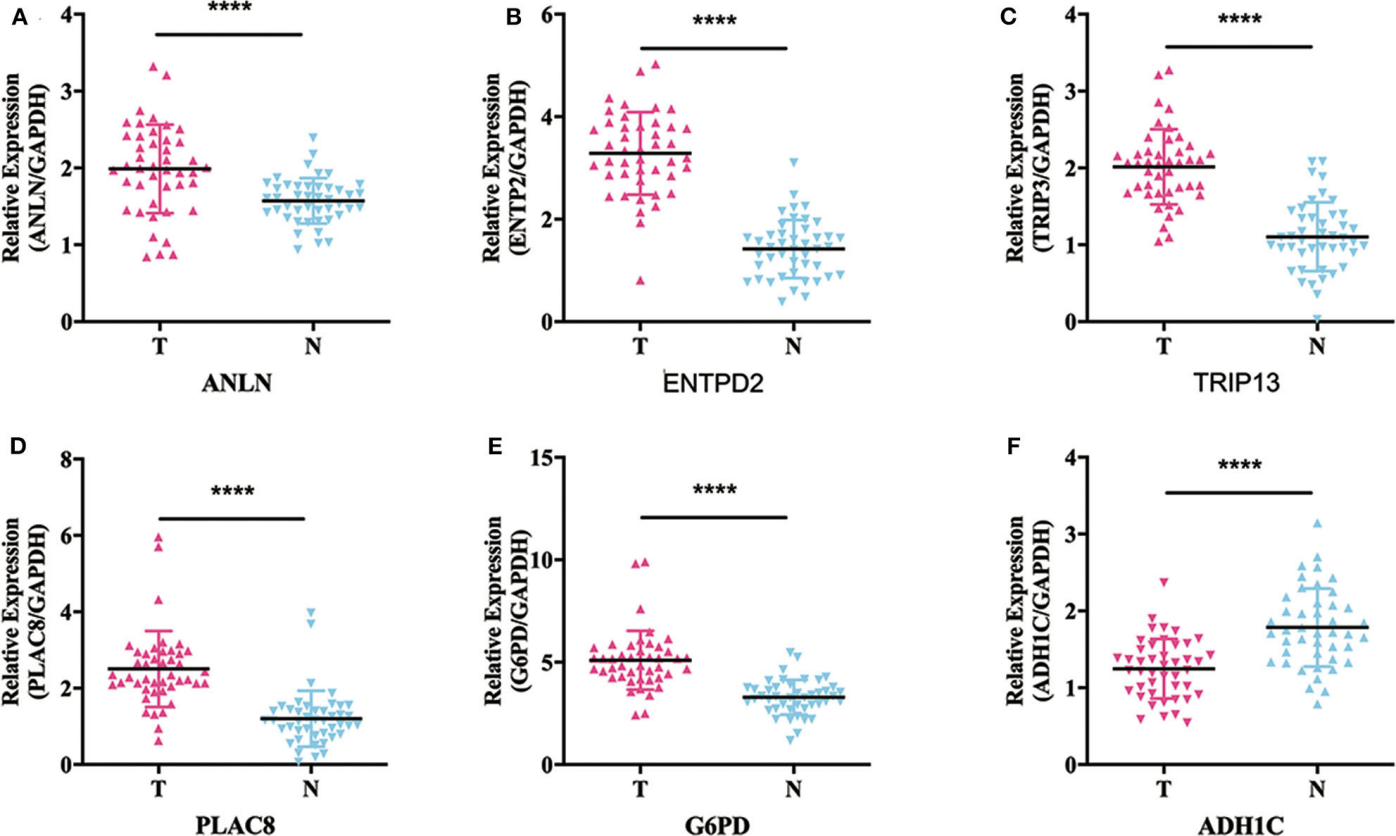
The results of qRT-PCR in six genes. **(A–E)** *ANLN, ENTPD2, TRIP13, PLAC8*, and *G6PD* were highly expressed in HCC tissues. **(F)** *ADH1C* was lowly expressed in HCC tissues.

**Figure 7:**
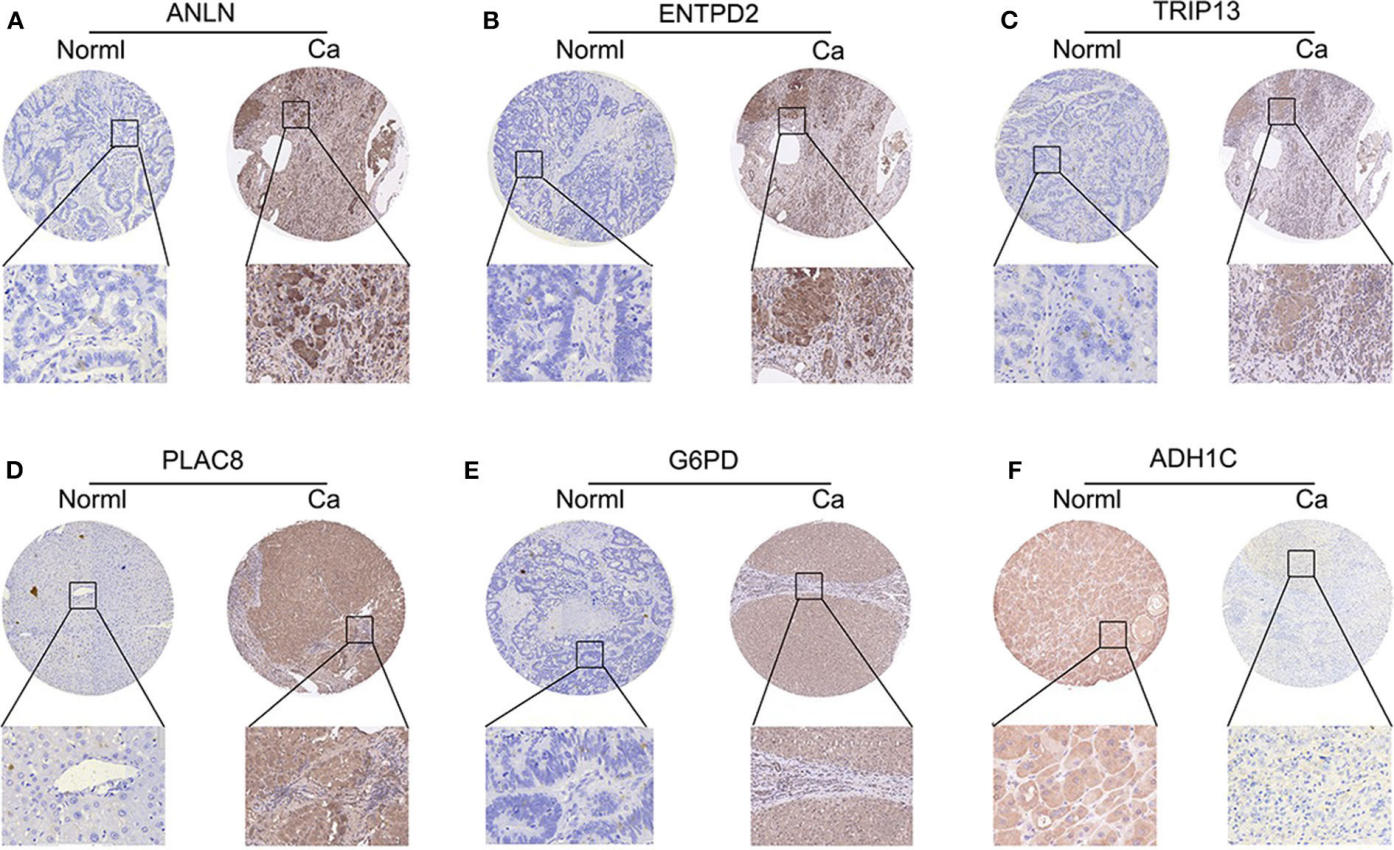
The results of immunohistochemistry in six genes. **(A–E)** *ANLN, ENTPD2, TRIP13, PLAC8*, and *G6PD* were highly expressed in HCC tissues. **(F)** *ADH1C* was lowly expressed in HCC tissues.

To validate whether *ANLN, ENTPD2, TRIP13, PLAC8*, and *G6PD* are highly expressed and *ADH1C* are lowly expressed in HCC tissues, we experimentally validated the expression in the tissues of 44 HCC patients. The results of qRT-PCR (Figure 6) and immunohistochemistry (Figure 7) suggested that *ANLN, ENTPD2, TRIP13, PLAC8*, and *G6PD* were highly expressed and *ADH1C* was lowly expressed in HCC tissues.

### Internal and External Validation to Evaluate the Robustness of the Risk Model

**Figure 8:**
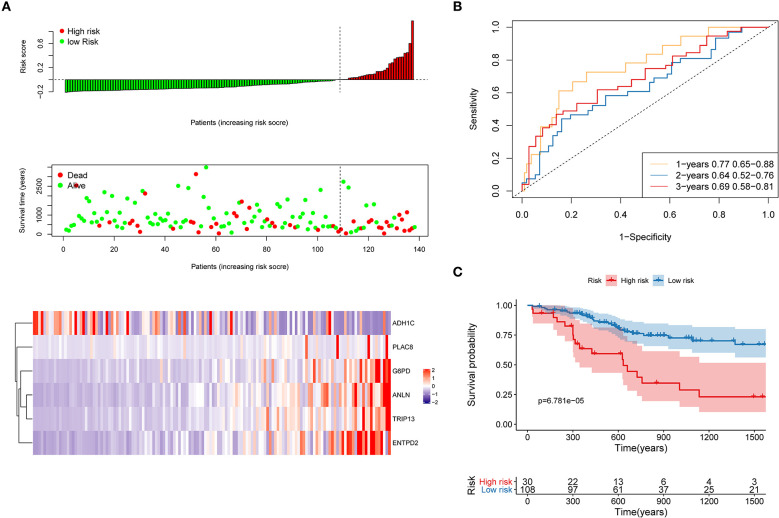
Internal validation of the risk model. **(A)** Distribution of risk score, OS, survival status (red dots indicate death, blue dots indicate alive) and the six genes expression heat map in the internal validation cohort; **(B)** ROC curves and AUC for 1-, 2-, and 3-year survival in the internal validation cohort of the risk model; **(C)** KM curves of the OS in the internal validation cohort.

First, the TCGA internal validation cohort was used to verify the robustness of the risk model that was established above (Figure 8). Figure 8A shows that high-risk score patients had worse OS which was in line with that of the training cohort. Furthermore, ROC curves were generated to analyze the efficiency of prognosis prediction for 1, 2, and 3 years in the internal validation cohort, as shown in Figure 8B. The model has a relatively high AUC that was above 0.66. According to the Gordon index (cutoff = 0.2280976), patients were divided into two groups with high and low risk. The OS of the two groups were compared using the KM curves (Figure 8C). The patients from the high-risk group had significantly worse survival outcomes compared with those from the low-risk group (*P* < 0.001).

**Figure 9:**
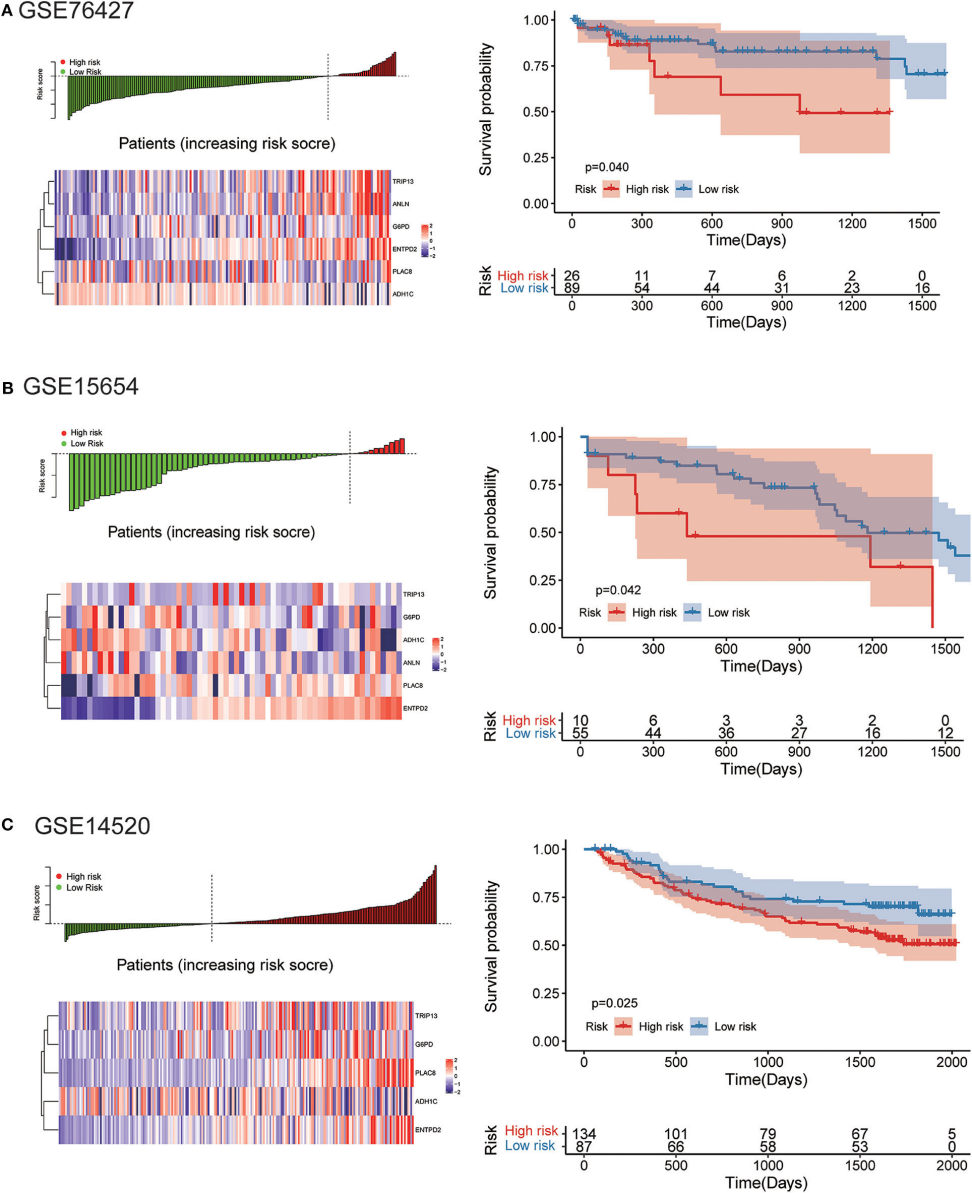
External validation of the risk model in GSE76427, GSE15654, and GSE14520 datasets. **(A)** The distribution of risk score, six genes expression heat map, and the KM curves of the OS in the GSE76427 validation cohort. According to the risk score, the patients were divided into the high-risk group and low-risk group in the GSE76427 dataset; **(B)** The distribution of risk score, six genes expression heat map, and the KM curves of the OS in the GSE15654 validation cohort. According to the risk score, the patients were divided into the high-risk group and low-risk group in the GSE15654 dataset; **(C)** The distribution of risk score, six genes expression heat map, and the KM curves of the OS in the GSE14520 validation cohort. According to the risk score, the patients were divided into the high-risk group and low-risk group in the GSE14520 dataset.

The robustness of the risk model was further be assessed with three external datasets using the GEO datasets of **GSE76427**, **GSE15654**, and **GSE14520** (GSE14520 only contains five genes without ANLN), respectively. Similar results that high-risk score was related to worse OS were obtained in three cohorts, as shown in Figure 9. The model had a relatively high distinguishing ability of prognosis and could identify the high-risk group patients with worse survival results in three different cohorts. Significant differences were present between the two groups, with the *P*-values of 0.040, 0.042, and 0.025 in three cohorts, respectively. ROC curves showing the efficiency of prognosis prediction for 1-, 2-, and 3- years in the three external validation cohorts were illustrated in Figure S7.

### GSEA Enrichment in the High-Risk Group and the Low-Risk Group

A total of 342 samples from the **TCGA-LIHC** dataset were divided into high- and low-risk groups based on the risk model we had established. GSEA enrichment was used for the analysis of the significantly enriched pathways in the two groups. The GSEA enrichment is listed in Table S10. Four importantly enriched pathways of high-risk (cell cycle regulation, Wnt signaling pathway) and low-risk group (xenobiotics biodegradation, metabolism, and lipid metabolism) were illustrated (Figure S8).

### Nomogram Construction

**Figure 10:**
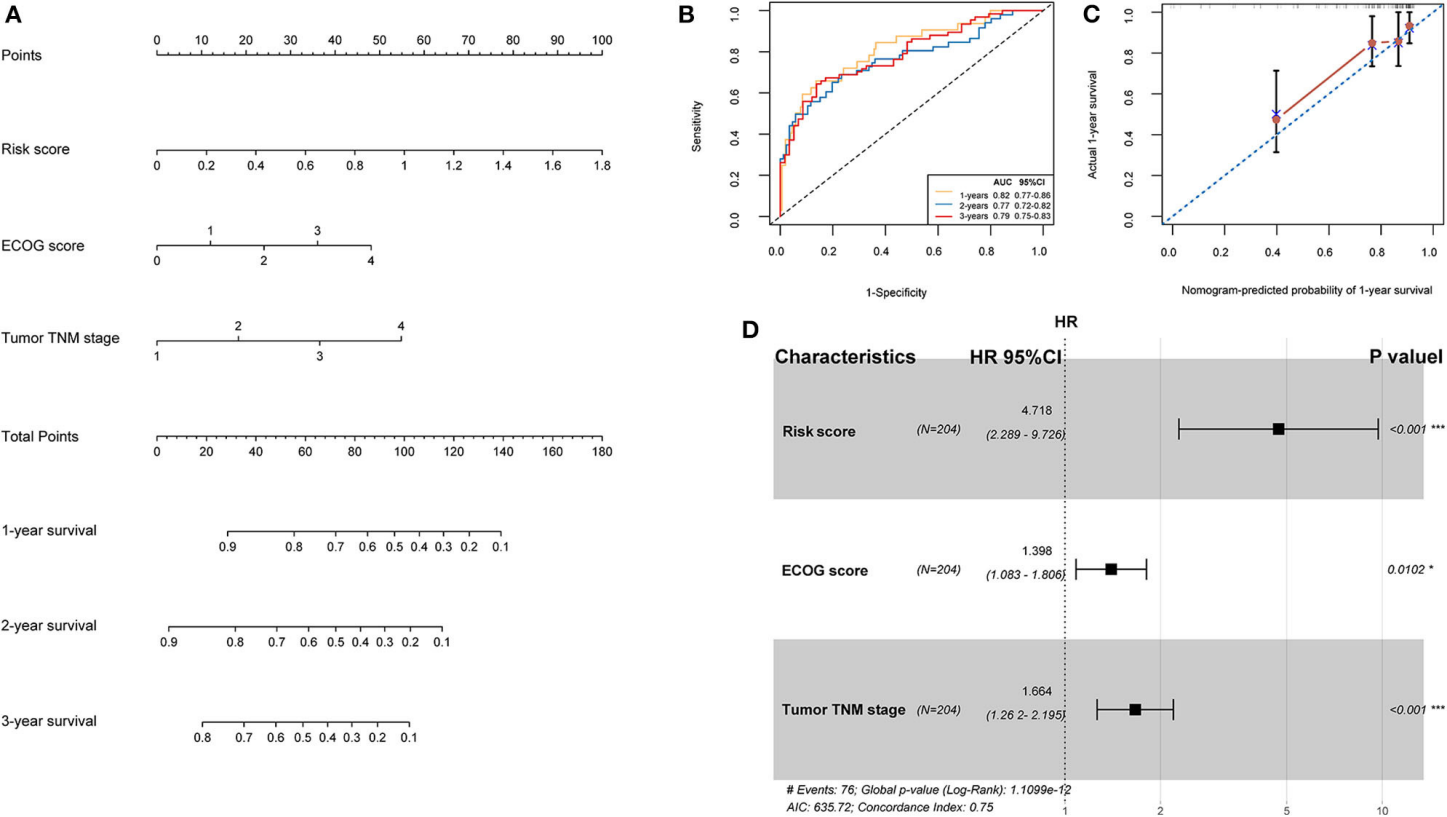
**(A)** Nomogram predicting the OS in HCC patients containing the risk score. **(B)** ROC curves and AUC for 1-, 2, and 3-year survival of the nomogram. **(C)** Calibration curve of 1-year survival in the nomogram and ideal model. **(D)** Forest plot of risk factors affecting the survival in HCC patients.

TCGA training cohort was used to screen the prognostic risk factors. The risk score, existing the inducement to HCC, hepatitis virus infection, a higher ECOG score, and tumor TNM stage were found to be risk factors for OS (Table 3) after the univariable Cox regression analysis. Then we made a multivariable Cox regression analysis using the above factors and we found risk score, ECOG score, and tumor TNM stage were independent risk factors for OS. The hazard ration (HR) value of risk score was the largest with the *P* < 0.001. Then, a nomogram model with a C-index value of 0.746 (95% CI=0.714–0.777) containing the above clinical features and risk score was constructed, as shown in Figure 10A. Furthermore, the score for each patient was calculated according to the nomogram, and the prediction accuracy of the nomogram was assessed using the ROC curve. The results showed that the AUCs of the nomogram model in 1-, 2-, and 3- years were 0.82, 0.77, and 0.79, respectively (Figure 10B). Figure 10C shows the calibration curve of 1-year survival between the nomogram and the ideal model. Results show that the 1-year nomogram model was consistent with the ideal model, indicating that the accuracy of our model was relatively high. The forest plot was utilized to show the clinical features such as risk score, ECOG score, and the tumor TNM stage in the nomogram, as shown in Figure 10D. The HR of risk score is around 4.718 (*P* < 0.001).

**Table 3:** Univariable Cox regression of OS in the training cohort.

**Characteristics**	***P-*value**	**HR**	**Low 95% CI**	**High 95% CI**
Risk score	<0.001	7.725	4.339	13.754
Age	0.1336	1.015	0.996	1.034
Gender	0.3209	0.790	0.496	1.258
Inducement, Yes vs. No	0.044	0.620	0.389	0.988
Hepatitis virus infection, Yes vs. No	0.007	0.505	0.307	0.831
ECOG score	<0.001	1.929	1.533	2.427
Tumor TNM stage	<0.001	2.005	1.555	2.585
Gleason grade	0.269	1.192	0.873	1.628

*TNM stage, ACJJ 6^TH^ or 7^TH^ TNM stage; Inducement, factors associated with the occurrence of HCC; Hepatitis virus, Hepatitis B and/or hepatitis C viruses; ECOG, Eastern Cooperative Oncology Group*.

## Discussion

The prognosis of HCC patients usually is not good, because HCC is highly malignant and progresses rapidly. The majority of HCC patients have been in an advanced stage when they are diagnosed, which means that most of them are losing the chance of accepting liver resection. Moreover, the treatment of advanced liver cancer is quite difficult. Even if advanced HCC patients receive chemotherapy or molecular-targeted drug therapy, the effect is usually poor due to the selectivity, and toxicity of those drugs. With the advancement of tumor molecular biology, prognostic markers reflecting tumor progression at the molecular level may be beneficial to realize individualized survival predictions with better accuracy (9).

Recently, molecular prognostic markers have drawn more and more attention of researchers in the survival prediction of liver cancer (10, 11). In addition, some molecular markers vary with tumor progression so that we can use them to dynamically detect disease progression or changes in the prognosis of tumor patients. Moreover, compared with a single one marker, a panel of molecular markers will significantly increase the accuracy in reflecting the prognosis of HCC.

As we all know, energy metabolism is the basis for the proliferation and invasion of tumor cells. We are particularly interested in the exploration of the relationship between tumor metabolic genes and prognosis in HCC. We want to discover some molecular markers from tumor metabolic genes and make them be a panel of prognostic markers.

In the present study, the public gene expression data from TCGA and GEO databases were utilized to classify HCC patients into three molecular subtypes based on 594 energy metabolism-related genes, and significant differences in prognosis were found among the three subtypes. Then, 576 DEGs among the three subtypes were identified. GO and KEGG enrichment analysis showed that these DEGs were closely associated functionally with tumorigenesis and development. Furthermore, a prognostic risk model including six genes, which were selected by univariate Cox and LASSO-Cox regression, was established. The risk model that consisted of *ANLN, ENTPD2, TRIP13, PLAC8, G6PD*, and *ADH1C* was effective and stable to predict HCC patients' prognosis according to internal and external validations. Moreover, a nomogram that had a good prognosis prediction of survival was built. The risk score is an important risk factor of OS with the biggest HR value in the nomogram. The enriched pathways in the high- and low-risk group obtained by GSEA analysis were significantly associated with the tumorigenesis and progression of HCC, such as cell cycle, Wnt signaling pathway, drug metabolism cytochrome P450, and primary bile acid biosynthesis. Last, qRT-PCR and immunohistochemistry at tissue level was utilized to validate the mRNA expression levels of the six genes screened above, and significant differences were found in the expression of mRNA between tumor and adjacent normal liver tissues.

In this study, we found three independent subtypes of metabolism patterns in HCC, but there was no significant difference in the prognosis between C1 and C3 subtypes. Although C1 and C3 represented two types of metabolism patterns by the NMF algorithm, there is no certain relationship between the different metabolic patterns and the different prognosis in HCC. With the development of HCC, patients with above two different metabolic patterns had the same prognosis. We just used the public database to make a primary exploration of the metabolism patterns in HCC. C1 and C3 are two different metabolism patterns, but they will lead to the same survival outcomes in HCC, which is an amazing and interesting discovery. Further research needs to be explored in the future.

*ANLN* has been identified as a biomarker in a variety of human cancers. For example, knocking down *ANLN* could strongly inhibit the migration ability of breast cancer cells (12). Silencing the expression of *ANLN* could significantly inhibit the migration and invasion abilities of pancreatic ductal adenocarcinoma lines (13). Besides, the expression of *ANLN* in breast cancer cells played an important role in cell division, and the high expression of *ANLN* in nuclear was associated with poor prognosis in breast cancer patients; thus, *ANLN* could be used as a prognostic marker in clinical practice (14). Some researchers found that *ANLN* is a key candidate gene for cervical cancer and HBV-related HCC (15, 16). In the present study, our risk model contained the *ANLN* gene. High expression of *ANLN* was found to be associated with high risk and poor prognosis for HCC patients.

David et al. showed that hypoxia can induce the high expression of *ENTPD2* in cancer cells of HCC, leading to an increase in extracellular 5'-AMP (5'-Adenosine Monophosphate), which could promote the maintenance of myeloid-derived suppressor cells (MDSCs) by inhibiting the differentiation of MDSCs. Inhibition of *ENTPD2* could suppress the proliferation of cancer cells (17). Our study proved that overexpression of *ENTPD2* could increase the risk of survival in HCC.

Silencing *TRIP13* could act as a tumor suppressor for liver cancer, which inhibited cell growth and metastasis *in vivo* and *in vitro* experiments (18). Overexpression of *TRIP13* abrogated mitotic spindle checkpoint and induced proteasome-mediated degradation of *MAD2* in multiple myeloma mainly through the Akt pathway. *TRIP13* could be used as a biomarker for the development and prognosis of multiple myeloma (19). High expression of *TRIP13* could lead to an increase of aggression, treat-resistant in tumor cells, and enhance the repair of DNA damage. It could also promote false non-homologous terminal connections in head and neck cancer and induce chemical resistance (20). *TRIP13* might also be a potential target for the treatment of colorectal cancer (21, 22). The conclusions of the above studies are consistent with ours.

The Role of *PLAC8* in tumorigenesis is controversial. Some researchers showed that endogenous *PLAC8* promoted cell proliferation and tumor formation in lung cancer (23). *PLAC8* could also promote proliferation and inhibit apoptosis in breast cancer cells by activating the PI3K/AKT/NF- κB pathway (24). Interestingly, *PLAC8* can exert different biological effects depending on the cell environment (25, 26) so that HCC patients with a high level of *PLAC8* had a high-risk score and bad prognosis in our risk model.

Glucose 6-phosphate dehydrogenase (*G6PD*) is a rate-limiting enzyme in the pentose phosphate pathway. Previous studies have shown that elevated *G6PD* expression could promote cancer progression in many tumors. For example, Chen et al. found that high *G6PD* expression was a risk factor of poor prognosis for bladder cancer and *G6PD* expression level increased when the patients had advanced tumor stage. Patients with lower *G6PD* expression in tumor resection have a better survival rate compared with bladder cancer patients with higher *G6PD* expression (27). In colon cancer, the destroyed NADPH dynamic balance mediated by *G6PD* could enhance oxaliplatin-induced apoptosis of colon cancer cells through REDOX regulation; thus, *G6PD* could be a potential prognostic biomarker and a promising target for colon cancer treatment (28). In the present study, high *G6PD* expression would get a high-risk score with worse survival. This result was consistent with previous research results.

Aldehyde dehydrogenases are enzymes that are mainly involved in alcohol metabolism. Several genes can encode aldehyde dehydrogenases with different features. Coding variants, such as *ADH1B* and *ADH1C*, can actively encode alcohol dehydrogenase (ADH) enzymes so that alcohol (i.e., ethanol) can be transformed into acetaldehyde faster. These alleles will produce a protective effect when patients have a risk of alcoholism (29). Other researchers proved that the gene polymorphisms in *ADH1C* might change the risk of forming esophageal squamous cell carcinoma by regulating acetaldehyde metabolism and propensity to drink (30). In the present study, *ADH1C* was a protective factor in HCC patients. Many HCC patients were inferred to have a background of alcohol abuse; thus, *ADH1C* overexpression can enhance the ability to transform alcohol into acetaldehyde rapidly and indirectly protects liver function from damage due to alcohol and forming HCC.

The advantage of this study is that we have identified a prognostic feature by 6-gene signature that predicts one-, two-, three- year survival with relatively high AUC in both the training and the validation cohorts. Our study had some limitations. First, our model consisted of six genes and it is better to use a fewer number of genes for prognostic prediction in models. Second, the six genes were only validated by qRT-PCR and immunohistochemical staining in HCC tissues. Although the RNA-seq data of TCGA were of high quality, further experimental verification, such as chromatin immunoprecipitation is needed. Besides, the functions of these six genes in HCC need to be further studied *in vitro* and *in vivo* experiments. Last, more preclinical studies and prospective clinical trials are needed to confirm our findings.

## Conclusion

Taken together, our study found three energy metabolism molecular subtypes in HCC patients with different prognosis. A Six-gene signature-associated risk model with good performance of prognostic prediction was established. This model could be used as an independent prognostic evaluation index for HCC patients.

## Data Availability Statement

Publicly available datasets were analyzed in this study. This data can be found here: GEO database (http://ncbi.nlm.nih.gov/geo/).

## Ethics Statement

The studies involving human participants were reviewed and approved by the Ethics Committee of The Second Affiliated Hospital of Dalian Medical University. The patients/participants provided their written informed consent to participate in this study.

## Author Contributions

QC and FL: development of methodology, analysis, and interpretation of data. JX and LL: study concept and design and drafting of the manuscript. YG and GX: data collecting, analysis, and interpretation. JX and LL: critical revision of the manuscript for important intellectual content and administrative support. All authors read and approved the final version of the manuscript.

## Conflict of Interest

The authors declare that the research was conducted in the absence of any commercial or financial relationships that could be construed as a potential conflict of interest.

## Supplementary Material

Figure S1 Schematic diagram of the parameters of the non-negative matrix algorithm.

Figure S2 Immune scoring was performed based on sequencing data of the three molecular subtypes using TIMER (Tumor Immune Estimation Resource). **(A)** Lymphocyte B cell immune score among the three molecular subtypes; **(B)** CD4^+^ cell immune score among the three molecular subtypes; **(C)** CD8^+^ cell immune score among the three molecular subtypes; **(D)** Neutrophil cell immune score among the three molecular subtypes; **(E)** Macrophages cell immune score among the three molecular subtypes; **(F)** Dendritic cell immune score among the three molecular subtypes.

Figure S3 Volcano map of differentially expressed genes among three molecular subtypes. **(A)** Volcano map of differentially expressed genes between C2 and C1 subgroups. **(B)** Volcano map of differentially expressed genes between C2 and C3 subgroups. The red dots indicate genes that are up-regulated, and the blue dots indicate down-regulated genes.

Figure S4 Sample clustering result between C1 and C2 subtypes.

Figure S5 Sample clustering result between C3 and C2 subtypes.

Figure S6 Protein-protein interaction network for differentially expressed genes based on the STRING database. Using the fast greedy clustering algorithm of the STRINGdb tool to cluster the interaction network and finally, these genes could significantly cluster into 8 networks **(A–H)**.

Figure S7 External validation of the risk model in GSE76427, GSE15654, and GSE14520 datasets. **(A)** The distribution of survival status (red dots indicate death, blue dots indicate alive) and the ROC curves for 1-, 2-, and 3-year survival in the GSE76427 dataset; **(B)** The distribution of survival status (red dots indicate death, blue dots indicate alive) and the ROC curves for 1-, 2-, and 3-year survival in the in GSE15654 dataset; **(C)** The distribution of survival status (red dots indicate death, blue dots indicate alive) and the ROC curves for 1-, 2-, and 3-year survival in the in GSE14520 dataset.

Figure S8 GSEA analysis results in the high- and low-risk groups. Cell-Cycle and Wnt-signaling-pathway were highly expressed in the high-risk group. The Metabolism of Xenobiotics by Cytochrome P450 and the Primary Bile Acid Biosynthesis pathway showed low expression in the low-risk group.

Table S1 Metabolic pathways from Reactome database.

Table S2 Energy metabolism related genes.
