# Supplementary figures and images for: Identification of Energy Metabolism Genes for the Prediction of Survival in Hepatocellular Carcinoma
Source: Front Oncol. 2020 Aug 13;10:1210. doi: 10.3389/fonc.2020.01210 (PMC7438573; doi:10.3389/fonc.2020.01210)

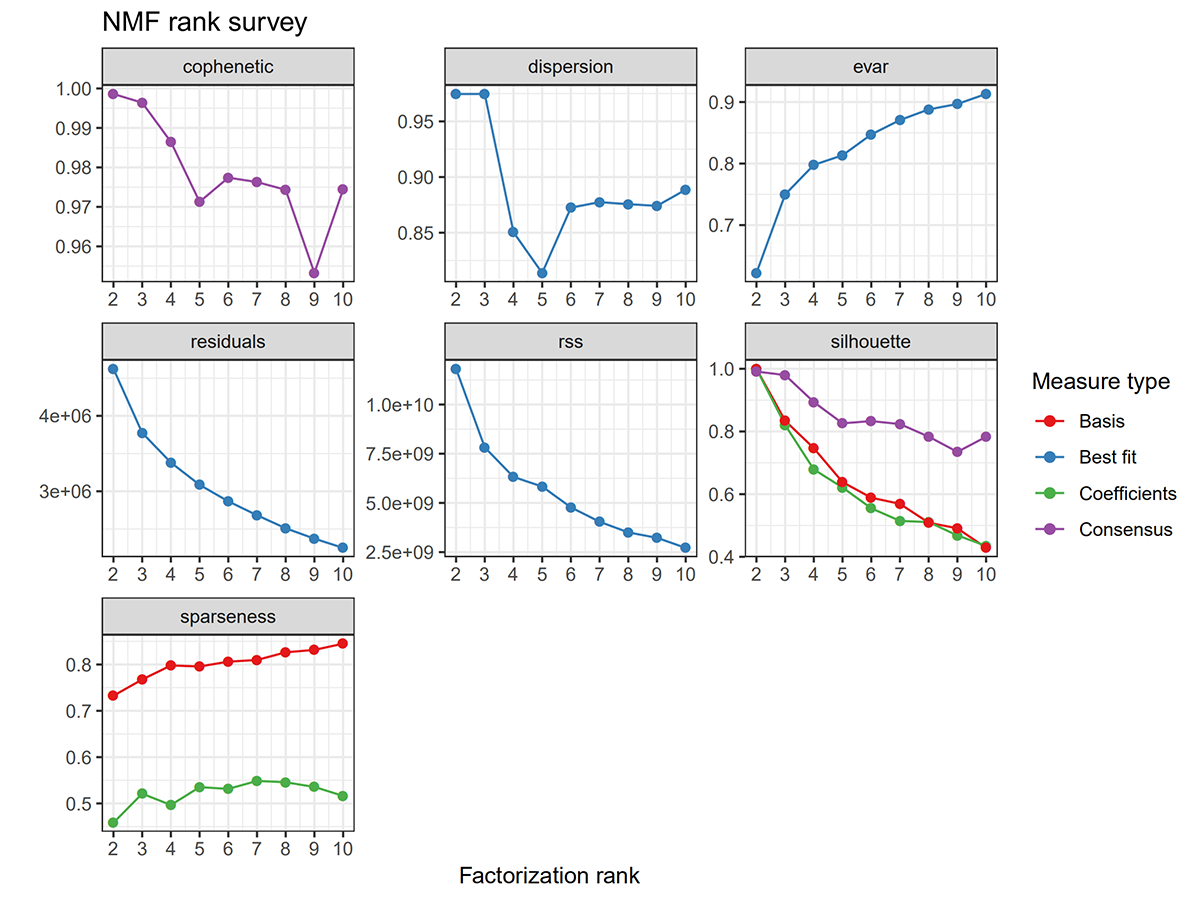

Supplement: Figure S1 — Schematic diagram of the parameters of the non-negative matrix algorithm. [file Image_1.TIF]

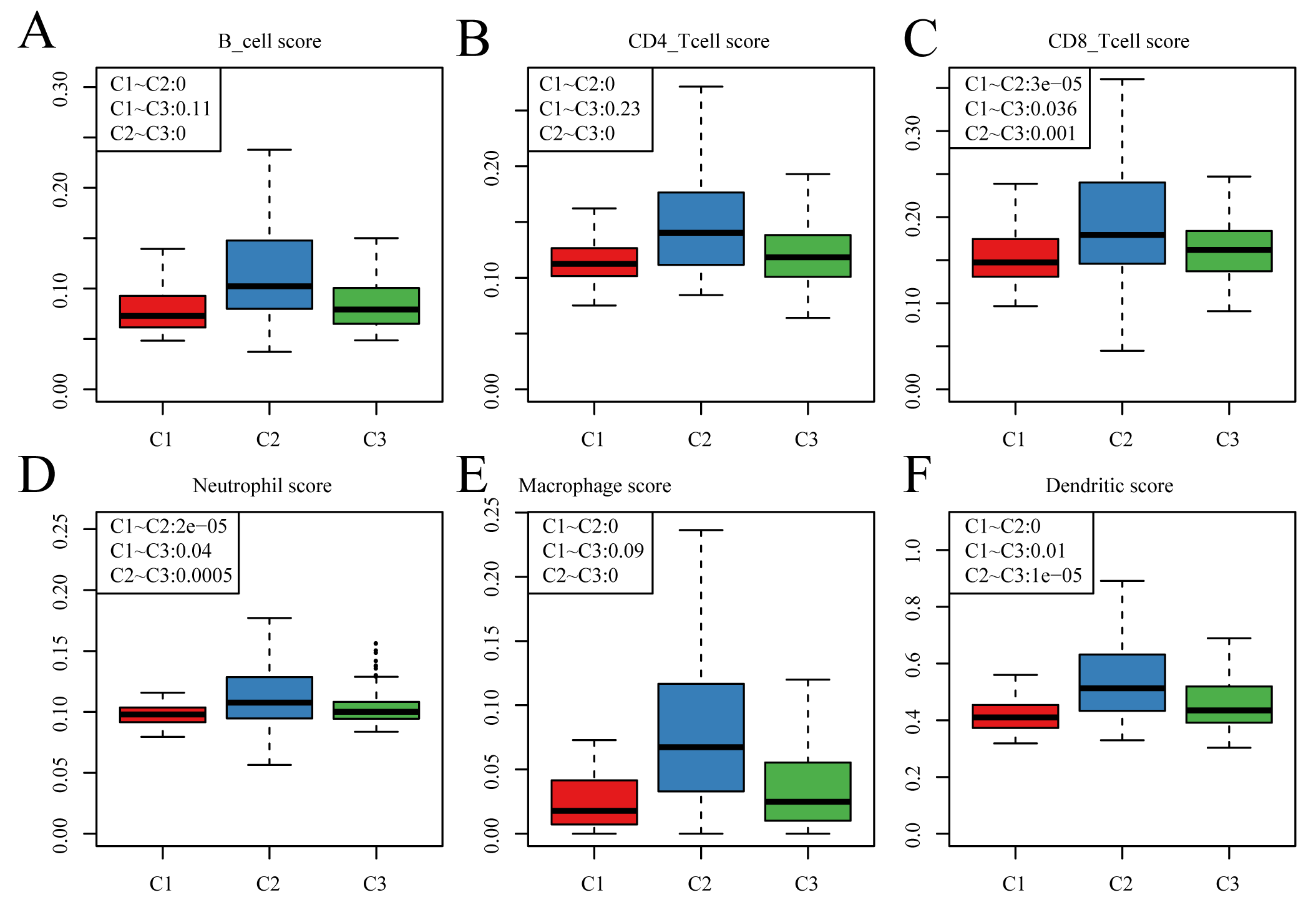

Supplement: Figure S2 — Immune scoring was performed based on sequencing data of the three molecular subtypes using TIMER (Tumor Immune Estimation Resource). (A) Lymphocyte B cell immune score among the three molecular subtypes; (B) CD4+ cell immune score among the three molecular subtypes; (C) CD8+ cell immune score among the three molecular subtypes; (D) Neutrophil cell immune score among the three molecular subtypes; (E) Macrophages cell immune score among the three molecular subtypes; (F) Dendritic cell immune score among the three molecular subtypes. [file Image_2.TIF]

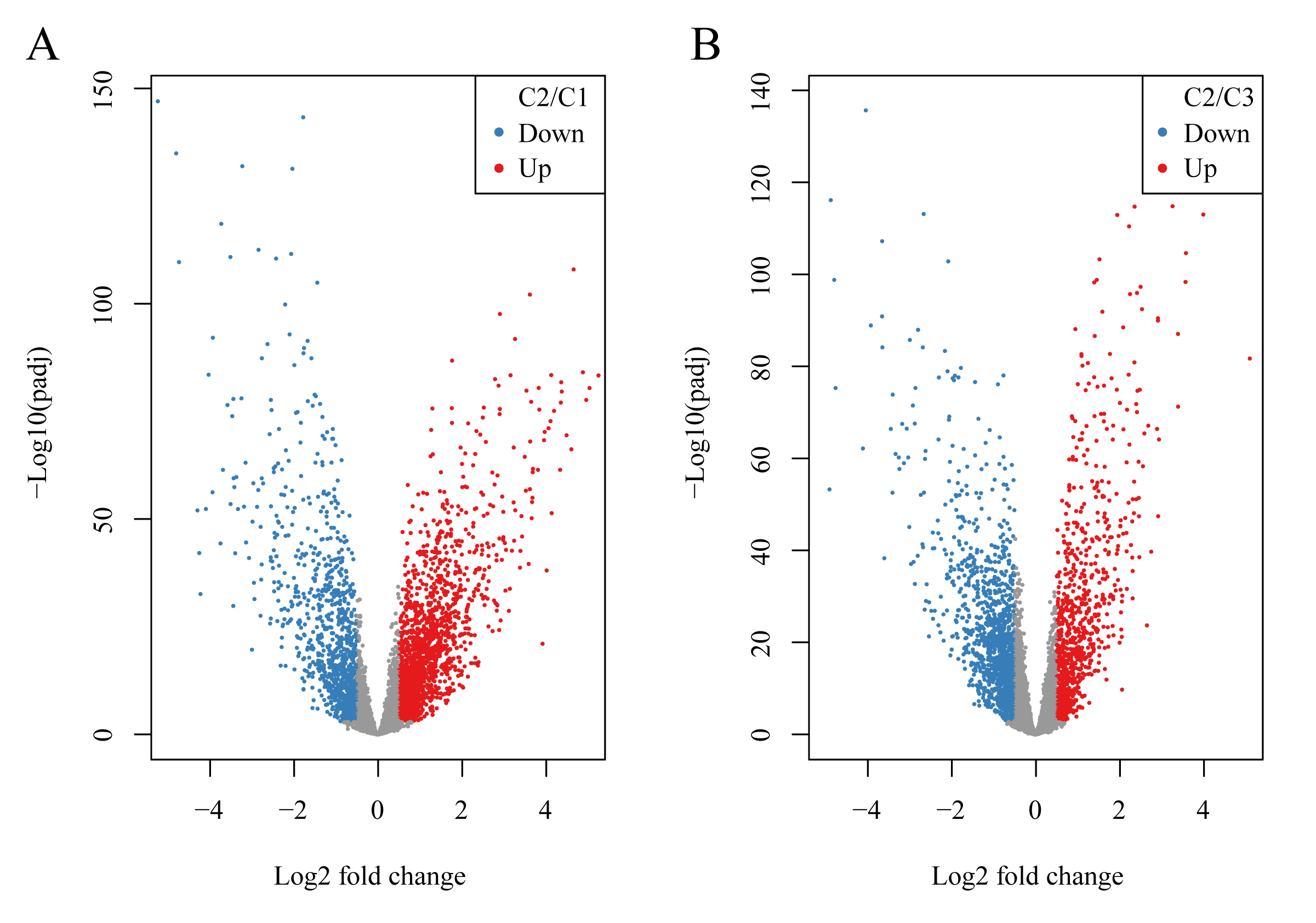

Supplement: Figure S3 — Volcano map of differentially expressed genes among three molecular subtypes. (A) Volcano map of differentially expressed genes between C2 and C1 subgroups. (B) Volcano map of differentially expressed genes between C2 and C3 subgroups. The red dots indicate genes that are up-regulated, and the blue dots indicate down-regulated genes. [file Image_3.TIF]

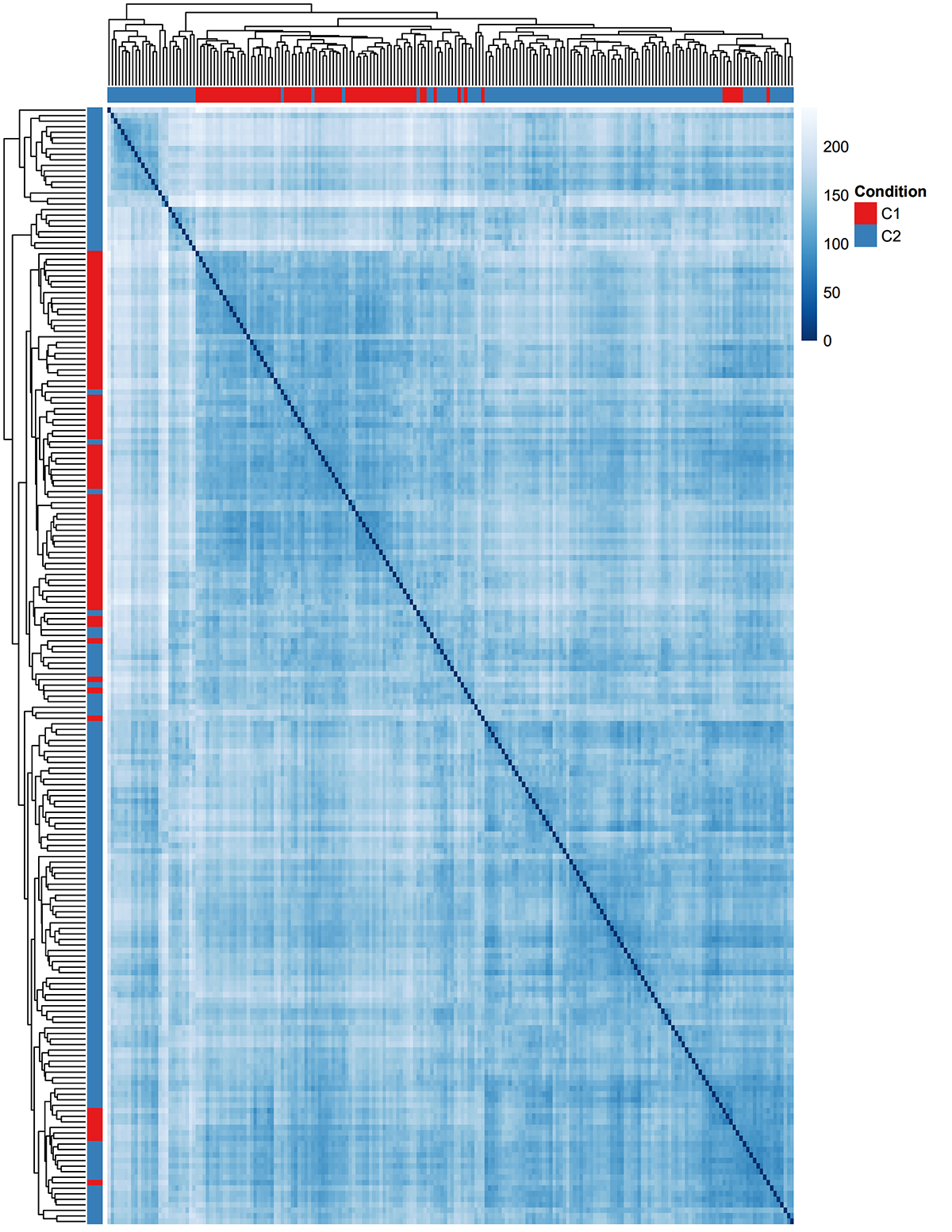

Supplement: Figure S4 — Sample clustering result between C1 and C2 subtypes. [file Image_4.TIF]

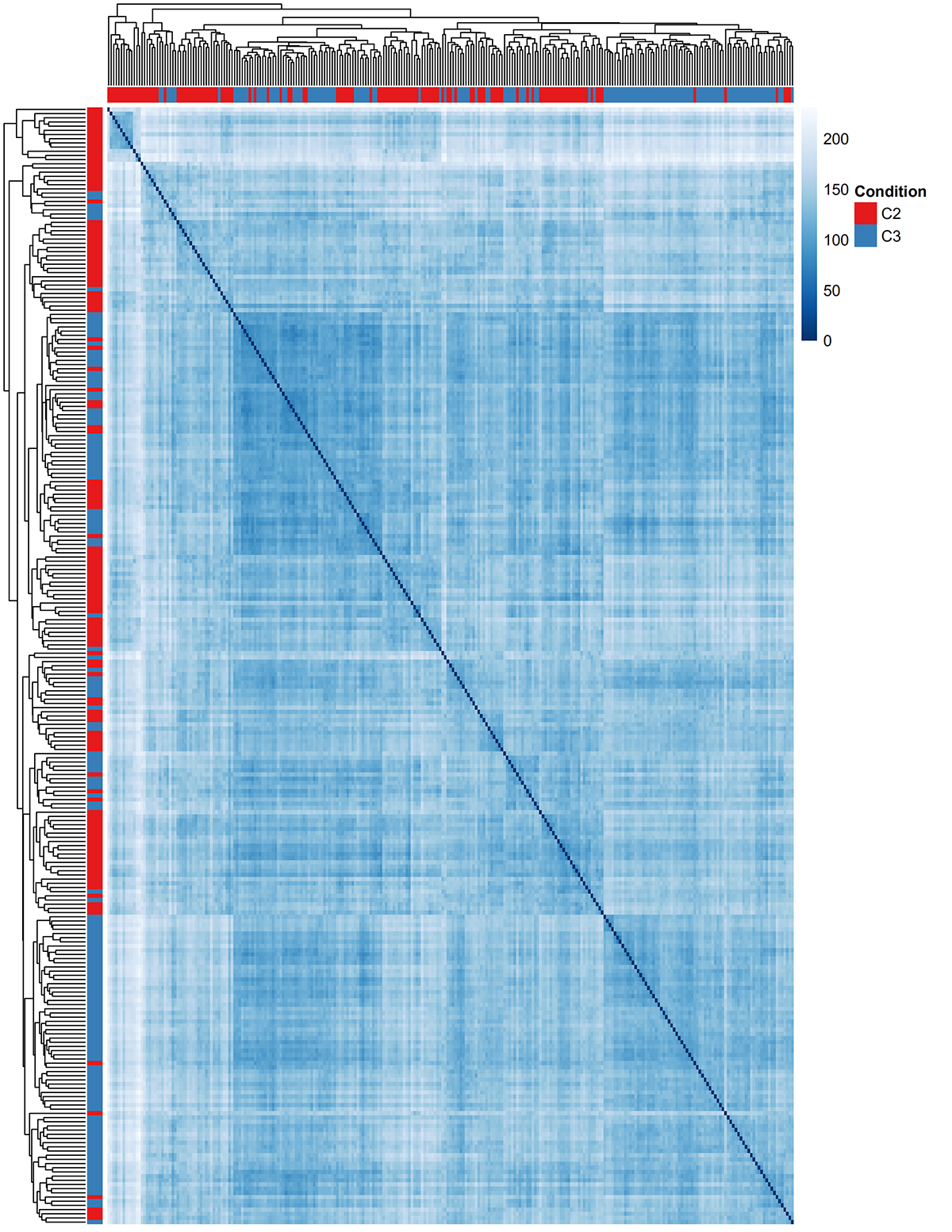

Supplement: Figure S5 — Sample clustering result between C3 and C2 subtypes. [file Image_5.TIF]

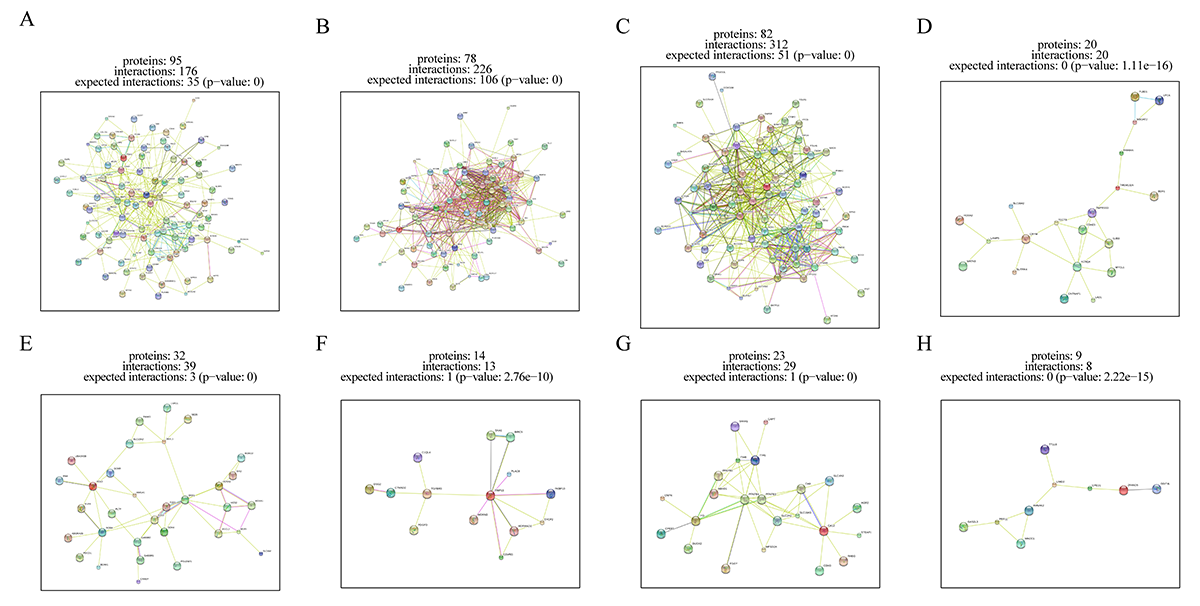

Supplement: Figure S6 — Protein-protein interaction network for differentially expressed genes based on the STRING database. Using the fast greedy clustering algorithm of the STRINGdb tool to cluster the interaction network and finally, these genes could significantly cluster into 8 networks (A–H). [file Image_6.TIF]

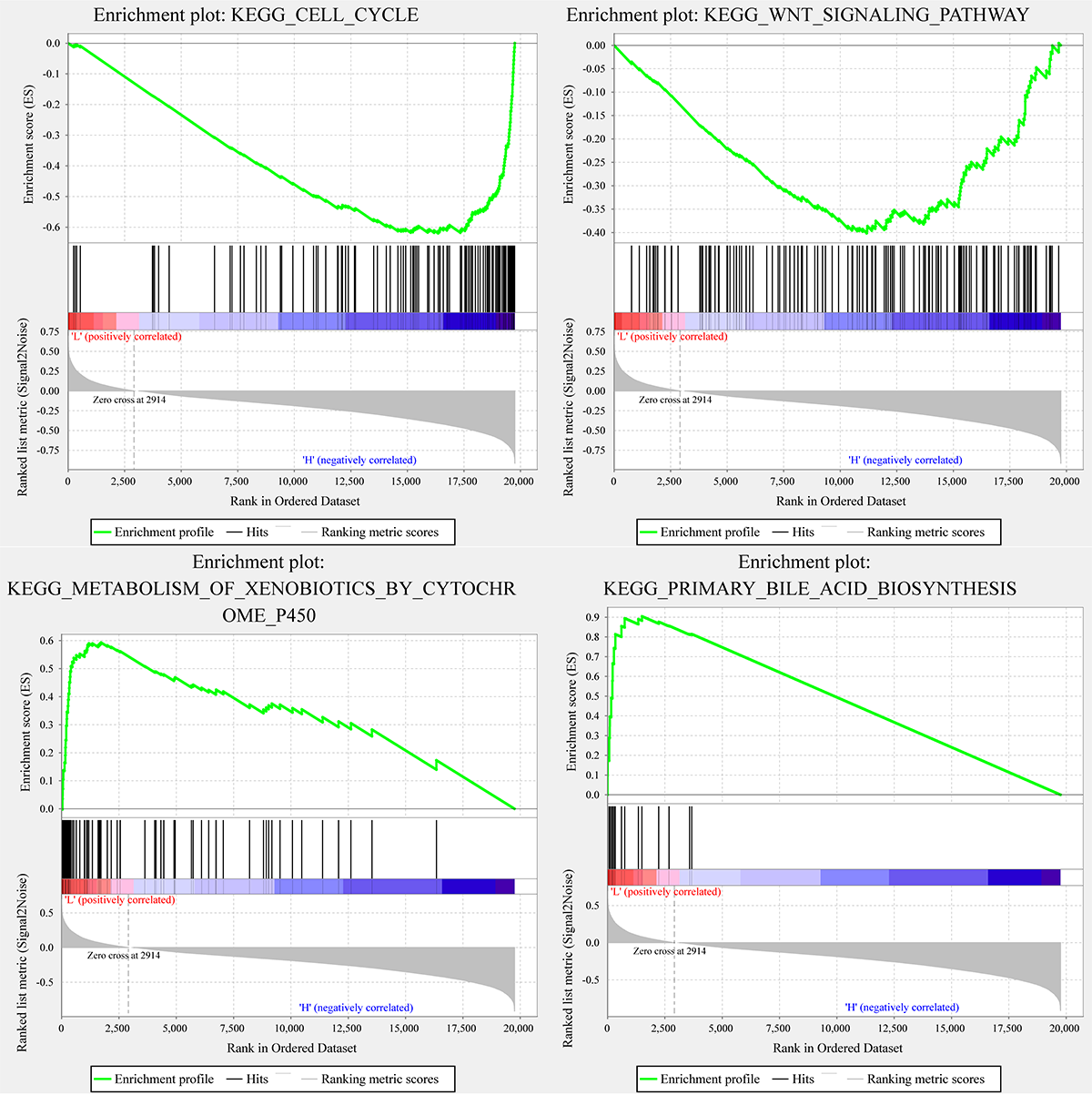

Supplement: Figure S8 — GSEA analysis results in the high- and low-risk groups. Cell-Cycle and Wnt-signaling-pathway were highly expressed in the high-risk group. The Metabolism of Xenobiotics by Cytochrome P450 and the Primary Bile Acid Biosynthesis pathway showed low expression in the low-risk group. [file Image_8.TIF]
